# Immunophenotypes and prognosis in autoimmune cardiovascular disease: a narrative review from clonal hematopoiesis to myocarditis, microvascular angina, and HFpEF

**DOI:** 10.1097/MS9.0000000000004901

**Published:** 2026-03-30

**Authors:** Suleman Khan, Fakhar e Mahin, Ibad Ur Rahman, Asifa Manzoor Ahmad, Areesha Nawaz, Zainab Ali Siddiqui, Aizaz Anwar Khalid, Hammad Iftikhar, Nawras Fashafsheh

**Affiliations:** a Department of Medicine, Khyber Medical College, Peshawar, Pakistan; b Department of Medicine, Fatima Jinnah Medical University, Lahore, Pakistan; c Department of Medicine, King Edward Medical University, Lahore, Pakistan; d Department of Medicine, Dow University of Sciences, Karachi, Pakistan; e Department of Medicine, Dow Medical College, Karachi, Pakistan; f Department of Medicine, Peshawar Medical College, Peshawar, Pakistan; g Department of Nursing, Al-Quds University, Jerusalem, Palestine, Department of Nursing, Faculty of Health Sciences, Istanbul Gelisim University, Istanbul, Turkey

**Keywords:** arrhythmias, autoimmune cardiovascular disease, cardiac autoantibodies, clonal hematopoiesis of indeterminate potential, coronary microvascular dysfunction, endothelial dysfunction, fibrotic remodeling, heart failure with preserved ejection fraction, immunomodulation, myocarditis, risk stratification, Th17/Treg imbalance, type I interferon

## Abstract

Autoimmune cardiovascular disease (CVD) includes myocarditis, coronary microvascular dysfunction (CMD), and heart failure with preserved ejection fraction (HFpEF). Traditional risk scores often overlook these conditions despite their links to high morbidity. Recent research highlights the significance of immunophenotyping, such as clonal hematopoiesis of indeterminate potential (CHIP), type I interferon activity, Th17/Treg imbalance, and cardiac autoantibodies, to improve risk assessment and guide treatment. This review combines mechanistic and clinical data connecting these immune signatures to endothelial dysfunction, arrhythmias, and fibrotic remodeling. It underscores the atherogenic role of CHIP and its influence on HFpEF prognosis, the vasculopathy driven by type I interferon signaling in CMD, and autoimmunity in myocarditis mediated by Th17 dominance. Cardiac-specific autoantibodies, like anti-heart and v1-adrenergic receptor antibodies, serve as early prognostic markers. Moreover, integrating immune biomarkers with advanced imaging techniques, such as cardiac magnetic resonance and positron emission tomography, could enhance risk stratification and therapy monitoring. Current immunomodulating treatments, including TNF and IL-6 inhibitors, provide cardiovascular benefits, while glucocorticoids and JAK inhibitors carry dose-related risks. In the future, personalized algorithms that incorporate CHIP genotyping, interferon scoring, and continuous biomarker and imaging assessments may transform autoimmune CVD management. This review highlights these translational insights and research priorities to bridge the gap between immune dysregulation and cardiovascular outcomes.

## Introduction

Autoimmune cardiovascular diseases such as myocarditis, microvascular angina, and ejection fraction reduced heart failure (HEpEF) pose a significant disease burden worldwide. According to a recent study, the overall risk of autoimmunity was 1.3–3.6 times higher for certain cardiovascular conditions like myocarditis, pericarditis^[^1^]^, heart failure^[^2^]^, and microvascular pathologies in individuals with existing autoimmune diseases compared to those without an autoimmune disorder. This is attributed to chronic inflammation and the powerful effects of autoantibodies on systemic circulation, which activate multiple signaling pathways^[^1^]^.

HIGHLIGHTS

- Immunophenotyping refines diagnosis across autoimmune myocarditis, CMD, and HFpEF.
- CHIP, IFN activity, and Th17/Treg imbalance are key determinants of CVD prognosis.
- Immune signatures map to endothelial injury, arrhythmias, and fibrotic remodeling.
- Integrating immune biomarkers with CMR/PET enhances risk and therapy assessment.
- Targeted immunomodulation shows benefit, while some agents carry dose-linked risk.

Systemic autoimmune diseases (SAD), such as Rheumatoid Arthritis (RA), Systemic Sclerosis (SS), and SLE, activate vascular and peri-vascular inflammatory pathways. A large number of CD4^+^ and CD8^+^ T cells are involved in the development of atherosclerotic CVD^[^3^]^. The prolonged release of proinflammatory markers, such as C-reactive protein, fibrinogens, and cytokines during chronic inflammation, along with autoantibodies targeting normal blood vessels, contributes to this process. These immune components infiltrate deeper into the tunica adventitia and tunica media, leading to endothelial damage, microvascular plaque formation, structural vessel abnormalities, and destabilization of the vessel wall, which increases mortality and morbidity^[^4,5^]^. The risk of cardiovascular diseases (CVD) in autoimmune diseases is often hidden by traditional or disease-specific risk factors such as age, sex, vessel wall characteristics, comorbidities, pre-existing atherosclerosis, and disease duration. There is no clear data on the use of existing cardiovascular risk scores, such as the Framingham Risk Score (FRS), QRISK3, or Systematic Coronary Risk Evaluation (SCORE), for assessing the severity or occurrence of CVD in autoimmune diseases^[^6^]^. Traditional risk scores offer little to no benefit in evaluating cardiovascular risk in autoimmune conditions and tend to underestimate the true disease burden.

Immunophenotyping has been effectively used as a therapeutic and prognostic marker in various autoimmune diseases. This review aims to synthesize evidence-based applications of immunophenotypes in specific cardiovascular conditions such as clonal hematopoiesis (CHIP), autoimmune myocarditis, microvascular angina/coronary microvascular dysfunction (CMD), and HFpEF. The review discusses the prognostic implications of disease-specific immune biomarkers (immunophenotypes, CHIP, cytokine profiles) and their therapeutic roles in calculating risk scores, severity scores, and overall disease management.

### Methodology

We conducted a targeted narrative review in PubMed/MEDLINE, Embase, and Google Scholar for studies published between January 2000 and May 2025. Keywords included immunophenotype, autoantibody, interferon, clonal hematopoiesis, myocarditis, microvascular angina, and HFpEF. We included mechanistic, observational, and clinical studies in English, prioritizing those relevant to autoimmune cardiovascular disease. The review followed SANRA (Scale for the Assessment of Narrative Review Articles) guidelines for transparency in search strategy and selection.

### Compliance with reporting guidelines

This article complies with the TITAN 2025 guidelines on declaration and use of AI tools in scholarly publishing^[^7^]^.

## Immunophenotypes in autoimmune CVD

### Clonal Hematopoiesis of Indeterminate Potential (CHIP)

#### Definition and prevalence

Clonal hematopoiesis of indeterminate potential (CHIP) is the presence of a clonally expanded hematopoietic stem cell caused by a leukemogenic mutation in individuals without evidence of hematologic malignancy, dysplasia, or cytopenia^[^8^]^. Almost 10–20% of individuals over 70 years old with CHIP are at increased risk of developing atherosclerotic CVD (ASCVD). Individuals with CHIP have a 0.5–1% increased risk of developing blood disorders such as leukemia. Secondary analysis of studies shows that individuals with CHIP have a 1.8–2% risk of developing coronary artery disease, a 4% risk of premature myocardial infarction, and a 2% risk of ischemic stroke in their lifetime, independent of traditional risk factors^[^8,9^]^. CHIP involves somatic mutations, also known as “DTA mutations,” in the driver genes DNMT3A, TET2, and ASXL1, which account for approximately 80% of the mutated hematopoietic stem cell growth in leukemias and ASCVD. These somatic mutations promote ASCVD by either increasing the chances of thrombosis or the risk of atherosclerosis by altering T-cell-induced immune pathways^[^9^]^.

#### Pathophysiology

**Figure 1.**
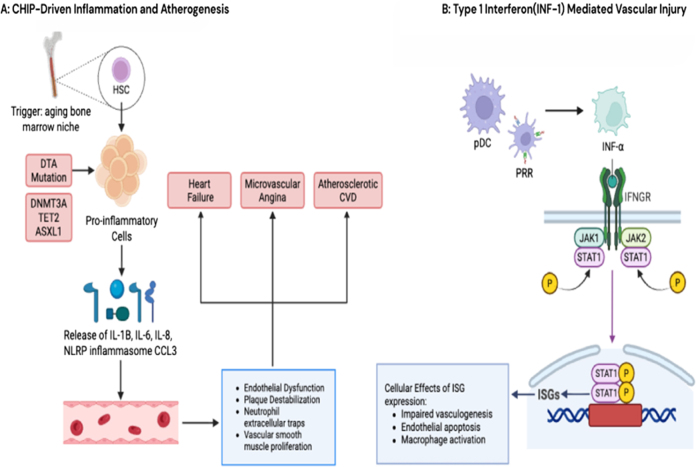
(A) Pathogenesis of CHIP and atherogenesis: it shows clonal expansion of aging hematopoietic stem cells that are subjected to somatic mutation (e.g., DNMT3A, TET2, ASXL1 genes). This gives the cells pro-inflammatory characteristics that cause release of cytokines like IL-1β, IL-6, IL-8, NLRP inflammasome and CCL3. Consequently, this cascade promotes endothelial dysfunction, plaque destabilization, neutrophil extracellular traps, and vascular smooth muscle proliferation which leads to heart failure, microvascular angina and atherosclerotic CVD. (B) Pathogenesis of IFN-mediated vascular injury: it shows production of IFN⍺ by plasmacytoid dendritic cells (pDCs), which activates the JAK-STAT signaling pathway and leads to transcription of IFN-stimulated genes, endothelial apoptosis, and macrophage activation, contributing to vascular injury.

Hematopoietic stem cells (HSCs) are more susceptible to mutations. The somatic mosaicism of CHIP develops with age, affecting cellular proliferation and viability over time. The DTA mutation involves changes in tumor-promoting genes that influence all cell lineages involved in mutant hematopoietic stem cell (HSC) expansion and self-renewal. It promotes pro-inflammatory states through the release of cytokines and inflammasome complexes. A cascade of inflammatory interleukins, such as IL-1, IL-5, IL-8, NLRP3 inflammasome, macrophage inflammatory proteins CCL3 and CCL5, and resistin protein, induces the expression of the CD58 gene. Additionally, JAK2 gain-of-function mutation leads to increased TET2 gene expression, linking extracellular signals to hematopoietic cell pathways. This results in endothelial dysfunction and thrombosis due to increased monocyte expression and adhesion to blood vessel walls, expanded formation of neutrophil extracellular traps (NETs), vascular smooth muscle proliferation, and plaque destabilization, which can cause angiotensin-induced cardiac hypertrophy, atherosclerotic plaque formation, reduced cardiac function, and increased cardiorenal fibrosis. These changes contribute to the development of microvascular angina and heart failure^[^8–12^]^. The detailed mechanism of CHIP and its role in atherogenesis is presented in Figure 1 (A and B), which illustrates the clonal expansion of hematopoietic stem cells, driven by somatic mutations, and the resulting pro-inflammatory effects leading to endothelial dysfunction, plaque destabilization, and vascular smooth muscle proliferation, contributing to microvascular angina and atherosclerotic cardiovascular disease.

#### Prognosis

CHIP has emerged as a risk factor for individuals at risk of developing cardiovascular diseases. It strongly correlates with cardiovascular events and the aging process. Studies show a close association of CHIP with the development of ASCVD, ischemic and non-ischemic etiologies, leading to reduced survival in patients with pre-existing HF. Individuals with DTA mutations have been shown to have worsened HF, with increased hospitalizations and deaths that are independent of age, sex, ischemic, and other non-modifiable risk factors^[^13,14^]^.

#### Gaps and future directions

Studies have shown a positive link between CHIP and various autoimmune diseases such as Crohn’s disease, Rheumatoid Arthritis, and Vasculitis due to increased inflammatory pathways. However, there is limited research on the exact mechanisms involved in each disease and their long-term complications in individuals. There is also little evidence regarding disease assessment scores and the effects of specific immunomodulatory therapies in autoimmune diseases associated with CHIP^[^8,14,15^]^.

### Type I interferon signature and endothelial dysfunction

#### Type I IFN pathway

Interferon type 1 is an inflammatory mediator and the first line of defense in innate immunity. It plays a significant role in diseases such as Systemic Lupus Erythematosus (SLE), Rheumatoid Arthritis (RA), systemic sclerosis (SS), and chronic inflammatory conditions^[^16^]^. IFN-1 exerts its effects through multiple mechanisms: by establishing an antibacterial state, modulating natural killer (NK) cell responses, and influencing adaptive immunity in inflammatory states. It also affects NK immunity by supporting NK cell production, maintaining homeostasis, and forming memory responses^[^12^]^.

Interferon 1 is a family of 16 members, including 12 subtypes of IFNα, as well as IFNβ, IFNε, IFNκ, and IFNω. All of these are regulated by activating the JAK-STAT pathway. Pathogens are detected by Pathogen Recognition Receptors (PRRs), which activate specific transcription factors such as IF-3. These factors then downregulate multiple transcriptional pathways to produce IFN-1 in an autocrine and paracrine manner. IF-3 signals through heterodimeric IFNAR to initiate phosphorylation of STAT-1, STAT-2, and TYK2. These form a STAT1-STAT2-IRF9 tri-complex that binds to specific DNA sequences in the nucleus, activating IFN-stimulated genes (ISGs) via the synthesis of IFN-stimulated response elements (ISRE). ISGs produce cascades of proteins after post-translational and epigenetic modifications, which are involved in host defense mechanisms. During infectious, autoimmune, or chronic inflammatory states, IFNs enhance antigen presentation, inhibit inflammasomes, promote the release of pro-inflammatory cytokines, and activate innate lymphoid cells and T cells in the bone marrow. They also inhibit pathogen entry, replication, and duplication through multiple cellular pathways^[^16–20^]^.

Dysregulation of the IFN signaling pathway plays an important role in autoimmune and chronic inflammatory diseases. Dysregulated IFNs exert their pleiotropic effects by producing dendritic cells (DC), natural killer cells (NK), Th1 T cell differentiation, and the release of cytokines^[^21,22^]^. In SLE, plasmacytoid DCs are the main producers of IFN-1, which deposit in the blood, skin, and kidneys, and are closely associated with the binding to anti-RBP antibodies found in other autoimmune diseases such as RA, systemic sclerosis, and idiopathic interstitial pneumonia^[^22,23^]^.

#### Evidence of vascular damage

IFN1 is known to cause premature vascular damage in patients with existing SLE. It disrupts normal endothelial cell apoptosis and vascular repair mediated by erythropoietic progenitor cells (EPCs) and circulating myeloid cells (CACs). Dysregulated IFNs inhibit the JAK-STAT pathway through complex mechanisms, leading to endothelial damage, impaired vasculogenesis, and early plaque formation^[^24,25^]^. IFNalpha is a characteristic finding in the peripheral blood smear of patients with SLE, indirectly serving as an independent marker of atherosclerosis. It assesses the cardiovascular risk that cannot be identified by traditional risk scores^[^24,26–28^]^.

#### Clinical implications

Dysregulated IFNs cause irreversible endothelial damage, activation of macrophages and foam cells, and vascular injury, leading to premature atherosclerosis and increased cardiovascular risk in autoimmune diseases such as SLE, RA, and SS. Studies show that anifrolumab, a monoclonal antibody that blocks the Type I IFN receptor, inhibits the formation of the IFNAR heterodimer, thereby preventing downstream activation of ISGs and signaling pathways. It has demonstrated significant results in reducing neutrophil extracellular trap (NET) formation, macrophage activation, and modulation of cardiometabolic disease markers. It has also been shown to improve SLE vasculopathy in patients.

### T helper 17 /regulatory T cell imbalance

CD4^+^ T cells play an important role in the adaptive immune response. They perform various functions such as activating B cells, producing different T helper cell subtypes, regulating macrophage activity, and modulating the body’s immune responses. Pathogens are identified by antigen-presenting cells (APCs) and presented to naive CD4^+^ T cells, which then differentiate into various effector cell subsets including T helper type 1 (Th1), Th2, follicular helper T, Th17, and regulatory T (Treg) cells^[^29,30^]^.

Th17 cell differentiation is activated by two key regulators, i.e., IL-12 and IFN-gamma, whereas Treg cells are directly derived from naive CD4^+^ T cells. The Th17 subset exhibits a proinflammatory effect, while Tregs exhibit an antagonistic effect. Their development is closely interconnected, and the Th17/Treg balance is important in autoimmune conditions where they maintain self-tolerance, as well as the expansion and activation of autoreactive CD4^+^ effector T cell responses. The imbalance is easily disrupted in autoimmune conditions such as RA and SS^[^29–31^]^.

Th17 cells differentiate under the influence of the transforming growth factor β signaling pathway into two important pro-inflammatory cytokines, i.e., IL-17 and IL-22. These cytokines have receptors on fibroblasts and epithelial cells, which regulate the local tissue inflammatory response and exhibit enhanced antimicrobial properties^[^32^]^. Treg cells express CD25 and play a role in innate and adaptive immune system homeostasis by regulating B cells, NK cells, macrophages/phagocytes, and dendritic cells, and they secrete anti-inflammatory cytokines such as TGF-beta, IL-10, and IL-35. Gene polymorphisms in surface receptors of the Th17 subset, along with the loss or reduction of Treg cells in certain autoimmune conditions, inflammatory states, and aging, disrupt the Th17/Treg balance^[^29,33^]^.

#### Myocarditis

Myocarditis is one of the main causes of antibody-driven chronic dilated cardiomyopathy and death in young adults. Autoimmune Myocarditis is characterized by inflammation of the heart caused by both adaptive and innate immune responses. An imbalance in the Th17/Treg axis plays a crucial role in experimental autoimmune myocarditis (EAM)^[^34,35^]^.

Myeloid-derived suppressor cells (MDSC) are a heterogeneous population of cells of myeloid origin that consist of myeloid progenitors, immature macrophages, immature granulocytes, and immature dendritic cells. They have both immunological and non-immunological functions in healthy individuals. MDSC expand through the JAK-STAT pathway, a common pathway that activates the IFN1 and T helper cells^[^36^]^. In humans, MDSC express CD33 but lack expression of mature myeloid and lymphoblast cell markers. This explains the role of MDSC in suppressing T-cell responses and reducing immune tolerance, while MDSCs may serve as key players in inflammatory responses and the pathogenesis of various autoimmune diseases^[^34^]^.

**Figure 2.**
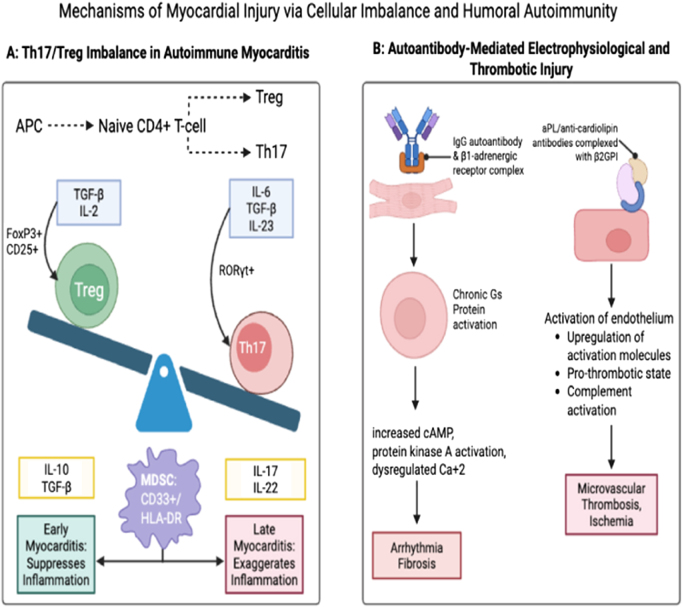
(A) Pathogenesis of T17/TReg Imbalance in Autoimmune Myocarditis: it shows dysregulated CD4^+^ cells that excessively differentiate into pro-inflammatory Th17 cells (driven by IL-6, TGF-β, and IL-23) secreting IL-17 and IL-22 (yellow box). It also shows downregulation in the differentiation of anti-inflammatory regulatory T cells (Tregs), which secrete IL-10 and TGF-β. Myeloid-derived suppressor cells (MDSCs) are context-dependent: early in the disease, they function as inflammation suppressors, while later in the disease, they function to exaggerate the response inflammation. (B) Pathogenesis of autoantibody-mediated electrophysiological and vascular injury: it shows (left) IgG autoantibody binding to the β1-adrenergic receptor on the cardiomyocyte. This leads to Gs-protein activation, which causes protein kinase A activation, increased cAMP, and dysregulated Ca^2^⁺. Consequently, arrhythmia and fibrosis develop. (Right) An aPL/anti-cardiolipin antibody binding with Beta-2-Glycoprotein I (β2GPI) on an endothelial cell. Subsequently, endothelial activation occurs, which upregulates adhesion molecules and creates a pro-thrombotic state via complement activation. As a result, microvascular thrombosis and ischemia occur.

In the early stages of EAM, MDSCs with selective depletion and adoptive transfer reduced local inflammation by decreasing the production of the Th17 cell subset. However, when MDSCs were transferred after selective depletion in late EAM, there was an exaggerated expression of IL-17 and FOX-3 receptors in CD4^+^ T cells, disrupting the Th17/Treg balance. This led to increased Th17 production and suppressed Tregs, resulting in severe myocardial inflammation. Another study using a mouse model shows that after myocarditis, myocytes exhibit increased Th17 cells and inflammatory cytokines. This indicates that Th17 cells display plasticity in the myocardium and contribute to the development of EAM^[^34,37^]^. The detailed pathogenesis of Th17/Treg imbalance in autoimmune myocarditis is presented in Figure 2(Slide A and B), which depicts the dysregulated differentiation of CD4^+^ T cells into pro-inflammatory Th17 cells, driven by cytokines like IL-6, TGF-β, and IL-23, as well as the reduced differentiation of regulatory T cells (Tregs), contributing to the inflammatory processes involved in myocarditis.

#### Clinical significance

Studies show that inhibiting mi-RNA 155 in EAM improves the balance of the Th17/Treg axis, indicating its potential therapeutic role in treating EAM^[^38^]^. Very little information is available on measuring circulating Th17 levels in patients with suspected or diagnosed EAM and on severity analysis. This represents a research gap, and further studies in this area are recommended.

### Autoantibodies and cardiac injury

#### Antiphospholipid and anti–β1-adrenergic receptor antibodies

Antiphospholipid antibodies and anti-cardiolipin antibodies have specific roles in endothelial cell injury due to their ability to bind with endothelial cell components through the beta 2 glycoprotein channel and activate the expression of intercellular adhesion molecule-1 (ICAM-1), vascular adhesion molecule (VCAM), endothelin-1, and P-selectin^[^39–41^]^. This activates processes leading to arterial and venous thrombosis, involving increased neutrophil infiltration, monocyte and platelet aggregation, vasospasm, and thrombus formation. aPL and lupus anticoagulants also activate pro-thrombotic agents and Annexin-1 molecules, which are involved in endothelial cell apoptosis, resulting in microvascular damage^[^41–43^]^.

Anti–β1-adrenergic receptor antibodies and anti-muscarinic antibodies alter the electrophysiology of heart muscles and are a leading cause of sudden cardiac death in young individuals^[^44^]^. β1-adrenergic receptors are G-protein-linked channels that activate the cAMP pathway, phosphorylate PKA, increase intracellular calcium influx from sarcoplasmic reticulum, and thus raise heart contractility and heart rate^[^45^]^. Studies have shown increased IgG autoantibody levels of anti–β1-adrenergic receptor antibodies in patients with inappropriate sinus tachycardia^[^46^]^. Studies also demonstrate a significant association between circulating anti B1-receptor autoantibodies, anti-M1 receptor antibodies, IL-6, and non-valvular AF. This indicates the role of these antibodies in autoimmunity and inflammation by altering the structural and electrophysiological properties of the heart^[^47^]^.

#### Anti-heart and anti-intercalated disc antibodies

Autoimmune myocarditis in certain autoimmune conditions such as systemic sclerosis, sarcoidosis, lupus, rheumatoid arthritis, vasculitis, or hypereosinophilia shows a high arrhythmogenic burden and poor prognosis^[^48,49^]^. Serum anti-heart (AHA) and anti-intercalated disk antibodies (AIDA) are organ- and disease-specific early markers of isolated (i.e., organ-specific) and autoimmune myocarditis^[^50,51^]^. Studies show a similar relationship of AHA/AIDA in autoimmune myocarditis due to higher cardiac specificity and cross-relation of AHA with cardiac muscles. AHA binds strongly to the alpha and beta myosin heavy chain isoforms in individuals with previous chest pain and increased troponin levels. This indicates myocarditis rather than pre-existing coronary artery disease^[^50^]^. Studies also suggest that positive AHA levels are associated with increased mortality during follow-up and cardiac deterioration over time. This makes it a strong predictor of mortality in individuals with autoimmune myocarditis^[^49–51^]^.

#### Prognostic potential

Autoantibody profiling in patients with myocarditis has shown positive results in identifying specific antigens involved in the disease’s cause^[^52,53^]^. This provides an important foundation and a diagnostic tool for individuals suspected of having myocarditis. However, no studies or guidelines currently recommend its use as a standard diagnostic method. While existing evidence indicates a link between cardiac autoantibodies and myocarditis, the exact mechanisms behind their pathogenic role are still unclear, emphasizing the need for long-term studies that combine immunological profiling with clinical outcomes.

## Clinical phenotypes and prognosis

### Autoimmune myocarditis

Myocarditis refers to the clinical and histological features resulting from a wide range of pathological immune responses affecting the heart^[^54^]^. Its incidence varies among different autoimmune diseases and is observed in patients with SLE^[^55^]^, rheumatoid arthritis^[^56^]^, dermatomyositis^[^57^]^, and ANCA-associated vasculitis^[^58^]^. In SLE, myocardial dysfunction is often multifactorial, linked to immune injury, ischemia, or comorbidities. Although myocarditis is clinically observed in only a small number of SLE patients, autopsy studies have shown a significantly higher incidence, indicating that the disease is often subclinical^[^55^]^. Myocarditis has been reported in 4–30% of rheumatoid arthritis cases. Dyspnea is the most common symptom in patients with RA and myocarditis. Histologically, two forms are seen in RA: the interstitial form, which is more commonly associated with systemic lupus erythematosus, and the granulomatous form, which is more specific to RA^[^56^]^. Myocarditis is a rare complication of dermatomyositis, believed to result from inflammation similar to that in skeletal muscle, leading to focal fibrosis, vasculitis, and vascular changes such as intimal proliferation and medial sclerosis^[^59^]^. In ANCA-associated vasculitis, myocardial involvement typically remains localized, most often affecting the basal part of the septum^[^58^]^. The pathogenesis of autoimmune myocarditis involves autoreactive T-cells and autoantibodies mediating injury to cardiac myocytes^[^60^]^.

### Prognostic markers

The prognostic markers of autoimmune myocarditis usually include cardiac autoantibodies, proinflammatory cytokines, and troponin levels. The Th17/Treg ratio can be used to assess disease activity. In experimental autoimmune myocarditis, an imbalance in this ratio was observed due to increased Th17 cells and decreased Treg cells, during both the initiation and progression of the disease. This imbalance occurred because of IL-6 mediated Th17 differentiation, which contributed to inflammation of the myocardium and subsequent fibrotic changes^[^35,61^]^. Autoantibodies, especially anti-heart antibodies and anti-intercalated disc antibodies, are specific early serological markers of autoimmune myocarditis. They can be used to detect both symptomatic patients and asymptomatic family members at risk^[^50^]^. Cardiac troponin is also an important diagnostic and prognostic marker; its levels indicate the degree of necrosis and severity of inflammation^[^62^]^.

### Management

Treatment typically involves the use of high-dose corticosteroids, while other anti-inflammatory therapies for myocarditis include NSAIDs, colchicine, monoclonal antibodies, and immunosuppressive agents such as azathioprine and methotrexate^[^63^]^. Emerging therapies include IL-17 inhibitors and JAK inhibitors, which directly target the immune mechanisms involved in the pathogenesis of myocarditis^[^64,65^]^ and can be used in patients who do not respond to steroids, although they increase the risk of opportunistic infections.

### Microvascular angina and coronary microvascular dysfunction

Coronary microcirculation controls vascular resistance and maintains proper blood flow based on oxygen needs. Coronary microvascular dysfunction describes a variety of conditions that decrease coronary flow reserve, potentially causing cardiac ischemia and angina even without obstructive disease in the epicardial coronary arteries. CMD can involve impairments in both endothelium-independent vasodilation and endothelium-dependent vasodilation.

### Evidence in autoimmune diseases

The incidence of CMD is common in patients with autoimmune diseases. Endothelial dysfunction, along with increased arterial stiffness, are the main causes of microvascular dysfunction. Increased levels of proinflammatory cytokines in SLE and other autoimmune diseases lead to decreased NO release and increased reactive oxygen species levels, which can cause endothelial dysfunction^[^66,67^]^. A 5-year follow-up study in SLE patients suggested that recurrent chest pain and adverse cardiovascular events, initially attributed to coronary artery disease, are actually due to CMD^[^68^]^. Importantly, traditional risk prediction tools, like Framingham scores, fail to predict CVD risk in patients with SLE and other systemic autoimmune diseases, highlighting the need for disease-specific assessment strategies^[^69^]^.

### Prognosis

CMD is linked to an increased risk of major adverse cardiovascular events such as heart failure, MI, and mortality compared to those with normal microvascular function^[^70^]^. It occurs more often in women than men and is associated with poor clinical outcomes^[^71^]^.

### Imaging and diagnosis

The diagnosis of CVD requires advanced techniques to reduce the risk of adverse cardiovascular events and mortality. Positron Emission Tomography (PET) and Cardiac Magnetic Resonance (CMR) are commonly used noninvasive tools for evaluating patients with suspected coronary microvascular dysfunction^[^72^]^. However, noninvasive imaging techniques indirectly measure the CFR by assessing myocardial blood flow at rest and during stress, while invasive techniques directly measure CFR and can be used to confirm the diagnosis.

### Heart failure with preserved ejection fraction in autoimmune diseases

Heart failure with preserved ejection fraction (HFpEF) makes up nearly half of all heart failure cases. Patients with HFpEF usually have a left ventricular ejection fraction (LVEF) of 50% or higher. Diagnosis is typically based on a combination of clinical examination, chest X-ray, ECG, and blood levels of biomarkers like BNP or NT-proBNP. HFpEF often develops either due to disruptions in left ventricular diastolic function or because of structural and functional changes in the left atrium.

### Autoimmune context

In patients with autoimmune disorders, the common heart failure type is HFpEF. In these patients, HFpEF often occurs without the influence of traditional cardiovascular risk factors or obvious ischemic heart disease. These patients usually exhibit diastolic dysfunction, microvascular impairment, and myocardial fibrosis, all detectable through cardiac imaging. Systemic inflammatory disorders specifically target the myocardial microcirculation, leading to endothelial dysfunction and fibrotic changes. Damage can happen directly through the coronary microvasculature or indirectly through inflammation-driven expansion and remodeling of epicardial adipose tissue. Since EAT shares an unobstructed microcirculation with the myocardium, it acts as both a source and amplifier of inflammation, worsening cardiac injury^[^73^]^.

### Role of clonal hematopoiesis

Clonal hematopoiesis (CH) is an immune-related process linked to various diseases, occurring when hematopoietic stem cells acquire somatic mutations. These mutations cause rapid growth of the mutated cells, which expand within the bone marrow and pass their mutations to daughter cells. This can alter immune cell function and contribute to a persistent, low-grade inflammatory state. The presence of clonal hematopoiesis in patients with HFpEF has been associated with poorer cardiac function, as reflected by adverse echocardiographic parameters and impaired diastolic function. Additionally, CH has been linked to worse long-term prognosis, demonstrated by a higher rate of cardiovascular-related hospitalization^[^74^]^.

### Prognosis and outcomes

The clinical course of autoimmune HFpEF is influenced by both systemic inflammation and specific immunophenotypes, both of which can lead to increased inflammatory markers. Systemic inflammation, triggered by obesity, hypertension, and insulin resistance, causes microvascular dysfunction, while immune cells can lead to ECM remodeling, which eventually results in myocardial stiffness^[^75,76^]^. Increased inflammatory markers in patients with HFpEF lead to exercise intolerance, higher mortality, and cardiovascular-related mortality^[^77^]^. However, a better understanding of the immunophenotypes of HFpEF can guide immunomodulatory treatments tailored to prognosis.

## Imaging and biomarkers for immunophenotype-driven risk stratification

### Imaging modalities

Cardiac imaging offers non-invasive insights into immune-mediated myocardial injury and remodeling. Cardiac Magnetic Resonance is a non-invasive tool that includes two main stress techniques: stress perfusion imaging and stress T1 mapping. Stress perfusion CMR involves analyzing first-pass gadolinium-enhanced images to detect regional perfusion abnormalities, especially in cases of single or two-vessel coronary artery disease. Stress T1 mapping is a contrast-free CMR method used to measure changes in tissue free water content.

Positron emission tomography is another well-established non-invasive technique that provides quantitative measurements of myocardial blood flow (MBF) and cardiac function at both rest and stress in a single scan^[^72^]^. FDG-PET is used to detect active inflammatory lesions in cardiovascular diseases and to monitor the effectiveness of anti-inflammatory treatments^[^78^]^. CXCR4-targeted PET is used for detecting cardiac remodeling and atherosclerosis^[^79^]^, and FAPI-PET is used to detect myocardial fibroblast activation^[^80^]^.

Computed tomography (CT) can estimate absolute myocardial blood flow using ECG-triggered imaging and mathematical modeling techniques such as arterial input function, upslope analysis, or deconvolution. Its high spatial resolution (0.5 mm) allows for detecting subendocardial perfusion gradients, and a reduced endocardial-to-epicardial attenuation ratio can indicate CMD^[^72^]^. CT also offers information about perivascular fat attenuation and can be used to assess cardiac inflammation^[^81^]^.

Echocardiography is another affordable, bedside-accessible technique. Myocardial contrast echocardiography uses contrast agents to measure myocardial blood flow^[^72^]^. It can be used to assess myocardial perfusion and blood flow.

### Biomarkers

In autoimmune cardiovascular inflammation, biomarkers can provide insights into the inflammation, immune activity, disease progression, and associated risks. Non-specific inflammatory markers linked to cardiovascular inflammation include C-reactive proteins, which can indicate the risk of cardiovascular disease when levels rise to 3 mg/l^[^82^]^. Glycoprotein acetylation (GlyA) is another inflammatory marker associated with cardiometabolic disease and mortality^[^83^]^. Interleukin-6 (IL-6) is linked to severe inflammation, myocardial fibrosis, and indicates injury to cardiac myocytes^[^84^]^. Soluble urokinase plasminogen activator (suPAR) reflects systemic immune activation and is associated with cardiovascular mortality and morbidity^[^85^]^. Myeloperoxidase, a reliable biomarker of myocardial infarction, enhances prediction of cardiac events when tested alongside CRP^[^86^]^.

Disease-specific biomarkers include type-1 interferon signatures, found in systemic and disease-specific autoimmune diseases, which are associated with endothelial dysfunction and major adverse cardiovascular events^[^87^]^. Autoantibodies like anti-heart and anti-intercalated disc antibodies are linked to myocarditis^[^50^]^.

Genetic risk factors, especially carriers of clonal hematopoiesis of intermediate potential (CHIP) mutations, are associated with poor cardiovascular outcomes and higher hospitalization rates^[^74^]^.

### Integration of imaging and biomarkers

Integrating imaging findings with circulating biomarkers can offer a more comprehensive view of disease activity in autoimmune cardiovascular conditions, helping physicians identify high-risk patients and refine treatment strategies. Experimental models show that biomarkers like IL-6 and glyA are associated with cardiovascular inflammation and myocardial fibrosis^[^84^]^. Advanced imaging techniques, especially cardiac MRI and PET, provide detailed insights into myocardial inflammation, fibrosis, and microvascular dysfunction^[^72,78–80^]^. These findings create a foundation for approaches where persistently elevated biomarkers of cardiovascular inflammation, combined with imaging evidence of microvascular inflammation or fibrosis, can guide clinicians in escalating immunotherapy. Conversely, normalization of biomarker levels and imaging findings may support safely tapering therapy. By combining immune biomarker data with advanced imaging, we can achieve a more accurate assessment of disease activity to guide treatment decisions and prognosis.

### Research gaps

Despite increasing interest in integrating biomarkers and imaging techniques, there is still limited knowledge about how changes in these measures over time relate to outcomes in conditions like autoimmune myocarditis, microvascular angina, and HFpEF. Most studies have examined these markers independently without tracking how they evolve as the disease progresses or responds to treatment. Consequently, due to the lack of long-term, disease-specific data, it is difficult to predict disease risk and determine whether a particular therapy is effective. This highlights the need for more research that not only links biomarkers and imaging techniques but also monitors disease progression over time, thereby improving treatment strategies for autoimmune cardiovascular diseases.

## Immunomodulatory therapy and cardiovascular outcomes

Immunomodulatory therapy, which includes a variety of medications such as TNF inhibitors, interleukin (IL)-17 and interleukin (IL)-23 inhibitors, NSAIDs, and JAK inhibitors, shows a wide range of cardiovascular (CV) outcomes^[^88^]^. However, a study indicates that taking tumor necrosis factor inhibitors (TNFi) over six months is associated with a 12% reduction in the risk of cardiovascular events (HR: 0.88, 95% CI: 0.81–0.95), with cumulative reductions reaching up to 51% over three years^[^89^]^. Similarly, a meta-analysis found that TNFi decreases the risks of myocardial infarction (MI) (RR: 0.81) and stroke (RR: 0.69)^[^90^]^. Furthermore, TNFi also help achieve remission in rheumatoid arthritis (RA), psoriatic arthritis (PsA), or spondyloarthritis (SpA), which is linked to reduced rates of serious CV events, primarily through suppression of inflammation^[^91^]^.

In contrast, interleukin-6 receptor blockade with tocilizumab appears to have a favorable CV safety profile, with the fewest major adverse cardiovascular events (MACE) compared to other biologics, and a lower risk of MI versus abatacept (HR: 0.67, 95% CI: 0.47–0.97)^[^92^]^. The ENTRACTE trial reported no significant difference in MACE between tocilizumab and etanercept (HR: 1.05, 95% CI: 0.77–1.43). Nonetheless, studies have shown that tocilizumab improves endothelial function, decreases arterial stiffness, and modulates oxidative and thrombo-inflammatory pathways, indicating possible vascular benefits beyond lipid changes^[^93^]^. Conversely, glucocorticoid therapy is strongly linked to a dose-dependent increase in CVD risks, with a meta-analysis of over 15 million individuals revealing an elevated risk of MACE (RR: 1.27), coronary heart disease (RR: 1.25), and heart failure (RR: 1.92) with greater exposure. Uncertainty regarding MACE also rises by 10% with each additional gram of cumulative glucocorticoid dose or by 63% with an extra 10 μg daily intake^[^94^]^. Additionally, cohort data show that even low daily doses (<5 mg prednisolone-equivalent) raise all-cause CVD risk (HR: 1.74), with a steep dose-response relationship at higher doses^[^95^]^. Moreover, in systemic lupus erythematosus, the total glucocorticoid dose closely correlates with an increase in the Framingham CV risk score, indicating a direct link to atherosclerotic burden^[^96^]^.

However, emerging safety concerns about the Janus Kinase (JAK) inhibitors, especially tofacitinib, have resulted in FDA warnings after reviewing increased MACE and thromboembolic risks compared to TNF inhibitors in RA patients with CVD comorbidities.

## Discussion

Immune dysregulation in autoimmune cardiovascular disease (CVD) manifests in distinct yet interconnected ways across myocarditis, microvascular angina (CMD), and heart failure (HF) with preserved ejection fraction (HFpEF). Therefore, in autoimmune myocarditis, an imbalance between Th17 and regulatory T cells triggers myocardial inflammation, fibrosis, and a subsequent transition to dilated cardiomyopathy^[^34,35^]^. Additionally, cardiac autoantibodies serve as important prognostic biomarkers that correlate with adverse outcomes and arrhythmogenic risk^[^50^]^. However, in CMD, diminished nitric oxide availability, oxidative stress, and type I interferon-derived vascular injury often mediate endothelial dysfunction^[^66,87^]^. Research indicates that HFpEF in autoimmune disease reflects chronic low-grade inflammation, often associated with clonal hematopoiesis of indeterminate potential (CHIP), which is accompanied by increased myocardial stiffness, diastolic dysfunction, and worse outcomes^[^74,77^]^. Despite these phenotypic differences, endothelial injury, maladaptive interferon signaling, Th17 imbalance, and pathogenic autoantibodies suggest that these conditions share a common spectrum of immune-mediated cardiac injury^[^3,13^]^.

However, the current literature has several limitations that hinder the assessment of dynamic immunophenotypic changes^[^68^]^. Additionally, the reported patient populations are diverse, as systemic lupus erythematosus (SLE), rheumatoid arthritis (RA), and systemic sclerosis each have distinct cardiovascular trends, making evaluation difficult^[^97^]^. Moreover, studies use circulating cytokines, imaging markers, or tissue-infiltrating immune cells, which results in no standard framework for describing immunophenotypes^[^98^]^. Similarly, positron emission tomography and cardiac MRI, which offer insights into inflammation and fibrosis, are rarely combined with longitudinal immune monitoring^[^78^]^. Furthermore, clinical medications like TNF and IL-6 inhibitors have been shown to reduce major adverse cardiovascular events (MACE) by lowering the incidence of myocardial infarction (MI) and stroke^[^90,92^]^; in contrast, glucocorticoids and JAK inhibitors are associated with significant cardiovascular risks, highlighting the need for precise treatment guidelines^[^94,99^]^.

Therefore, future research focuses on prioritizing prospective, multifaceted cohorts that combine single-cell and spatial transcriptomics, proteomics, and metabolomics to explain distinct immunophenotypes across myocarditis, CMD, and HFpEF^[^100^]^. CHIP genotyping and type I interferon activity (which predict adverse prognosis regardless of typical risk factors) should be integrated into cardiovascular risk models^[^15,28^]^. Additionally, using imaging and biomarker monitoring, along with tracking longitudinal changes in IL-6, GlycA, suPAR, or cardiac autoantibodies, combined with cardiac MRI or PET imaging, could provide insights into therapy responsiveness^[^62,83^]^. Interventional studies are also recommended to assess whether baseline immune signatures (Th17/Treg ratio, interferon score) predict response to IL-17 blockade in myocarditis or interferon receptor antagonists in SLE-related CMD^[^38,101^]^.

Clinically, these findings emphasize the importance of cardiovascular screening in patients with autoimmune conditions. Classic risk scores, such as the Framingham or SCORE, effectively highlight the cardiovascular risk in patients with autoimmune diseases^[^69^]^. Furthermore, personalized immunomodulatory therapy tailored to the dominant immune pathway (such as IL-6 blockade for vascular dysfunction and IL-17 inhibition for myocarditis) represents the future of management. Therefore, this approach, along with cautious use of glucocorticoids and JAK inhibitors, offers hope for improving survival and reducing morbidity in autoimmune cardiovascular disease^[^12,91^]^.

## Conclusion

Autoimmune cardiovascular diseases such as myocarditis, coronary microvascular dysfunction, and HFpEF are part of a range of immune-driven heart injuries caused by clonal hematopoiesis, dysregulated interferon signaling, Th17/Treg imbalance, and harmful autoantibodies. These immune profiles not only contribute to endothelial dysfunction, myocardial fibrosis, and increased risk of arrhythmias but also serve as potential prognostic markers and treatment targets. Current evidence shows the limitations of traditional risk scores and emphasizes the need to incorporate immune signatures, advanced imaging, and biomarker monitoring to enhance risk assessment and guide immunomodulatory therapy. Customizing treatment based on the primary immune pathways – while reducing exposure to glucocorticoids and JAK inhibitors – offers a chance to improve outcomes, decrease morbidity, and personalize care for patients with autoimmune cardiovascular diseases.

## Data Availability

The data supporting the findings of this study are available from the corresponding author upon reasonable request.

## Assistance with the study

The authors declare that no additional assistance was received for the conduct of this study.

## Presentation

The authors confirm that no preliminary data from this study have been previously presented.
